# Potentially Inappropriate Medication Use and Hard Braking Events in Older Drivers

**DOI:** 10.3390/geriatrics6010020

**Published:** 2021-02-20

**Authors:** Yuqing Xue, Stanford Chihuri, Howard F. Andrews, Marian E. Betz, Carolyn DiGuiseppi, David W. Eby, Linda L. Hill, Vanya Jones, Thelma J. Mielenz, Lisa J. Molnar, David Strogatz, Barbara H. Lang, Tara Kelley-Baker, Guohua Li

**Affiliations:** 1 Department of Epidemiology, Mailman School of Public Health, Columbia University, New York, NY 10032, USA; yuqingxue95@gmail.com (Y.X.); TJM2141@cumc.columbia.edu (T.J.M.); 2 Department of Anesthesiology, Vagelos College of Physicians and Surgeons, Columbia University, New York, NY 10032, USA; stc2126@cumc.columbia.edu (S.C.); BL2309@cumc.columbia.edu (B.H.L.); 3 Department of Psychiatry, Vagelos College of Physicians and Surgeons, Columbia University, New York, NY 10032, USA; Howard.Andrews@nyspi.columbia.edu; 4 Department of Biostatistics, Mailman School of Public Health, Columbia University, New York, NY 10032, USA; 5 Department of Emergency Medicine, University of Colorado School of Medicine, Aurora, CO 80045, USA; MARIAN.BETZ@ucdenver.edu; 6 VA Eastern Colorado Geriatric Research Education and Clinical Center, Aurora, CO 80045, USA; 7 Department of Epidemiology, Colorado School of Public Health, University of Colorado Anschutz Medical Campus, Aurora, CO 80045, USA; Carolyn.DiGuiseppi@cuanschutz.edu; 8 University of Michigan Transportation Research Institute, Ann Arbor, MI 48109, USA; eby@umich.edu (D.W.E.); ljmolnar@umich.edu (L.J.M.); 9 Center for Advancing Transportation Leadership and Safety (ATLAS Center), Ann Arbor, MI 48109, USA; 10 School of Public Health, University of California San Diego, La Jolla, CA 92093, USA; llhill@ucsd.edu; 11 Department of Health, Behavior and Society, Johns Hopkins Bloomberg School of Public Health, Baltimore, MD 21205, USA; vjones@jhu.edu; 12 Center for Injury Science and Prevention, Columbia University Irving Medical Center, New York, NY 10032, USA; 13 Bassett Research Institute, Cooperstown, NY 13326, USA; david.strogatz@bassett.org; 14 AAA Foundation for Traffic Safety, Washington, DC 20005, USA; tkelleybaker@aaafoundation.org

**Keywords:** aging, driving safety, hard braking events, potentially inappropriate medications

## Abstract

Potentially inappropriate medications (PIMs) identified by the American Geriatrics Society should generally be avoided by older adults because of ineffectiveness or excess risk of adverse effects. Few studies have examined the effects of PIMs on driving safety measured by prospectively and objectively collected driving data. Data for this study came from the Longitudinal Research on Aging Drivers study, a multisite naturalistic driving study of older adults. Multivariable negative binominal modeling was used to estimate incidence rate ratios and 95% confidence intervals of hard braking events (proxies for unsafe driving behavior defined as events with a deceleration rate ≥0.4 g) associated with PIM use among older drivers. The study sample consisted of 2932 drivers aged 65–79 years at baseline, including 542 (18.5%) who used at least one PIM. These drivers were followed through an in-vehicle recording device for up to 44 months. The overall incidence of hard braking events was 1.16 per 1000 miles. Use of PIMs was associated with a 10% increased risk of hard braking events. Compared to drivers who were not using PIMs, the risk of hard braking events increased 6% for those using one PIM, and 24% for those using two or more PIMs. Use of PIMs by older adult drivers is associated in a dose-response fashion with elevated risks of hard braking events. Reducing PIM use in older adults might help improve driving safety as well as health outcomes.

## 1. Introduction

Drugs that may impair driving safety include illicit substances, such as cocaine and amphetamines, and prescription medications, such as opioids, benzodiazepines, and antidepressants [1]. Older drivers are particularly at heightened risk of crash involvement due in part to use of prescription medications for chronic medical conditions [2,3,4]. In addition, age-related decline in psychomotor skills and cognitive functioning may compromise driving ability among older adults [5]. 

The American Geriatrics Society (AGS) Beers Criteria [6] has identified three categories of potentially inappropriate medications (PIMs): (1) medications that should be generally avoided by older adults aged 65 years or older; (2) medications that are potentially inappropriate in older adults with certain disease conditions; and (3) medications that should be used with caution. Research on PIMs has focused on the first category of medications because these medications are deemed ineffective or harmful due to unfavorable risk-benefit ratios. PIMs most commonly used by older drivers are psychoactive medications, such as benzodiazepines, tricyclic antidepressants, first-generation antihistamines, and opioids [7]. Side effects of these medications include drowsiness, slowed reaction time, impaired attention, dizziness, confusion, and delirium [1,8,9,10]. A number of psychoactive medications have been individually linked to increased risks of crash involvement and crash culpability [1]. For instance, meta-analyses indicate that use of antidepressants is associated with a 40% increased risk of crash involvement [11] and that use of prescription opioids is associated with a 129% increased risk of crash involvement and a 47% increased risk of crash culpability [12]. 

Previous research on medication use and driving safety was conducted largely in middle age adults although the prevalence of medication use, particularly polypharmacy use, is much higher in older adults [13,14]. Moreover, few studies have assessed the relationship between medication use and driving safety based on objectively and prospectively collected driving data. Driving safety can be measured in different ways according to data availability, data quality, study objective, and other considerations. While commonly used safety outcome measures, such as crashes, injuries, fatalities, and violations, are readily available through administrative and surveillance systems, they are infrequent events and may not be sensitive enough to detect an adverse effect in a defined study sample within a reasonable timeframe. Moreover, accurate and detailed driving exposure data, such as driving patterns and driving space, are essential for rigorously measuring driving safety but are often lacking [15]. In recent years, the naturalistic driving design has been increasingly used in road safety research as it incorporates technologies, such as sensors, small cameras, and other recording devices, to capture detailed data on vehicle maneuvers (e.g., left turns and right turns, speed, and deceleration), driving patterns, performance, and behaviors [16]. Objectively measured data from naturalistic driving studies allow researchers to examine the effects of risk-taking behaviors, such as distracted driving, on real-world safety performance using surrogate indicators, such as hard braking events (i.e., near-crashes) [17]. The present study aims to examine the association between PIM use and the risk of hard braking events in a large cohort of older drivers with prospectively collected naturalistic driving data. 

## 2. Materials and Methods 

### 2.1. Study Sample

Data for this study came from the Longitudinal Research on Aging Drivers (LongROAD) project, an ongoing prospective cohort study of older drivers conducted in five sites (Ann Arbor, MI; Baltimore, MD; Cooperstown, NY; Denver, CO; and San Diego, CA) and sponsored by the AAA Foundation for Traffic Safety (Washington, DC). The LongROAD research team recruited 2990 older drivers from primary care clinics and health care systems. At the time of enrollment, study participants met the following inclusion criteria: (1) being 65–79 years of age; (2) holding a valid driver’s license; (3) being fluent in English; (4) driving at least once a week on average; (5) residing in the catchment area of any one of the five study sites for at least 10 months per calendar year; (6) having no plans to move outside of the catchment area within the next 5 years; (7) having access to a motor vehicle of model year 1996 or newer with an accessible on-board diagnostics II port; and (8) driving the primary vehicle ≥80% of the time if access to more than one vehicle. Those with significant cognitive impairment or degenerative medical conditions that may severely affect driving safety, such as Alzheimer’s disease and related dementias, were not eligible for enrollment. The study design and methods were described in detail elsewhere [18]; all participants completed informed consent and the study was approved by each site’s Institutional Review Board. Of the 2990 participants in the LongROAD study, 58 (1.9%) were excluded from this analysis due to missing baseline medication data (*n* = 41) or unavailable driving data (*n* = 17).

### 2.2. PIM Use

The exposure measure of primary interest in this analysis was PIM use, which was determined based on medication data collected at baseline through the “brown-bag review” method [18]. For the baseline in-person assessment, participants were asked to bring all currently used prescription and over-the-counter medications and supplements with them for review. Trained research staff completed a standard data form for each medication and supplement. Data on up to 50 medications and supplements were collected for each participant and then classified based on the pharmacologic therapeutic classification system established by the American Society of Health-System Pharmacists in the American Hospital Formulary Service (AHFS) Clinical Drug Information [19,20]. PIMs were identified according to the American Geriatrics Society 2015 Beers Criteria [6,7]. PIM use was first analyzed as a binary variable (yes/no) and then as a 3-level ordinal variable based on the number of PIMs identified (0, 1, or 2 and more). 

### 2.3. Hard Braking Event

The outcome measure in this analysis was hard braking event, defined as a braking event with a deceleration rate ≥0.4 g. Data on these events and other driving behaviors were captured during the follow-up through the OBDII DataLogger (Danlaw, Inc., Novi, MI, USA), a recording device installed in the study participant’s primary vehicle at the time of enrollment. As a surrogate measure for aggressive or unsafe driving behavior, hard braking events are widely used in naturalistic driving studies [17,21,22,23,24]. 

### 2.4. Statistical Analysis

Incidence rates of hard braking events per 1000 miles driven were computed according to demographic characteristics, urbanicity, and PIM use. Urbanicity was based on the Rural Urban Commuting Area values for zip codes of study participants and was defined as urban (core region of a metropolitan statistical area), suburban (noncore region of a metropolitan statistical area) or rural (nonmetropolitan area) [25]. Because of over-dispersion in hard braking events, multivariable negative binominal modeling was used to estimate incidence rate ratios (IRRs) and 95% confidence intervals (CIs) of hard braking events associated with PIM use with adjustment for confounding variables. Data analyses were performed using SAS version 9.4 (SAS Institute Inc., Cary, NC, USA).

## 3. Results

Of the 2932 drivers included in this analysis, 1374 (46.9%) were male, 1203 (41.0%) had graduate degrees, and 2134 (72.8%) lived in urban areas; average age at baseline was 71.08 ± 4.06 years. Use of at least one PIM was reported by 542 (18.5%) drivers (15.3% used only one PIM and 3.2% used two or more PIMs). Benzodiazepines were the most commonly used PIM, accounting for 16.6% of the PIMs identified. Other frequently used PIMs were non-benzodiazepine hypnotics (16.4% of the PIMs identified), antidepressants (15.2%), and first-generation antihistamines (10.5%). 

After enrollment, the study participants were followed through the DataLogger for up to 44 months. The follow-up accumulated a total of 65,870,870 miles and 76,414 hard braking events, yielding an incidence rate of 1.16 hard braking events per 1000 miles. The incidence rate of hard braking events varied by demographic characteristics (Table 1). Specifically, drivers who were 70 years and older, female, nonwhite, or not married had elevated incidence rates of hard braking events whereas those who had an annual household income ≥$50,000 or resided in suburban and rural areas had decreased rates of hard braking events (Table 1). The incidence rate of hard braking events for drivers using PIMs at baseline was significantly higher than for those not using PIMs (1.30 vs. 1.13, *p* < 0.01; Table 1). 

**Figure 1:**
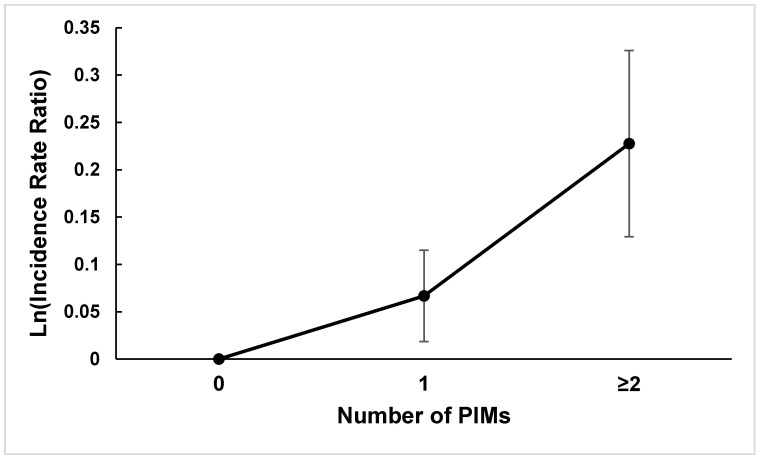
Adjusted incidence rate ratios of hard braking events and 95% confidence intervals according to the number of potentially inappropriate medications (PIMs) used by older drivers, the Longitudinal Research on Aging Drivers (LongROAD) Study.

The final multivariable negative binominal model included only those variables statistically significantly associated with the risk of hard braking events on the multivariate level (Table 2). With adjustment for race, marital status, and urbanicity, PIM use was associated with a 10% increased risk of hard braking events (adjusted IRR 1.10, 95% CI 1.01–1.20). When PIM use was categorized into three groups based on the number of PIMs used, the estimated IRRs of hard braking events showed a dose-response relationship, increasing from 1.06 (95% CI 0.97–1.17) for those using one PIM to 1.24 (95% CI 1.02–1.51) for those using two or more PIMs compared to drivers not using PIMs (Figure 1). When the analysis was stratified based on gender, the dose-response relationship between PIM use and the risk of hard braking events existed in both male and female drivers. In addition to PIM use, drivers who were nonwhite, not married or residing in urban areas were at significantly increased risk of hard braking events (Table 2). 

## 4. Discussion

PIM use in older adults has been linked to adverse drug reactions, excess healthcare costs, and increased morbidity and mortality [26,27,28,29]. This study is among the first attempts to assess the potential effect of PIM use on driving safety in older drivers. Results of this study indicate that PIM use is associated with a modest but statistically significant increase in the risk of hard braking events among older drivers. With adjustment for race /ethnicity, marital status and urbanicity, PIM use is associated with a 10% increased risk of hard braking events. Moreover, there exists a dose-response gradient in the relationship between PIM use and the risk of hard braking events.

Based on objectively and prospectively collected driving data, these findings add valuable evidence to the existent literature regarding the health and safety consequences of PIM use in older adults. Specifically, this study suggests that reducing PIM use through innovative intervention programs, such as computerized prescribing decision support tools [30], may help improve both health outcomes and driving safety among older adults. For older adults, driving is an important determinant of autonomy, independence and health [18,31]. Nevertheless, multiple chronic medical conditions (e.g., arthritis and cardiovascular disease) and polypharmacy use are prevalent in older drivers. Over 90% of the LongROAD study participants at baseline had two or more chronic medical conditions (with a median of 5 chronic medical conditions) and were on two or more medications (with a median of 7 medications) [20,32]. Of the medications used by older drivers, PIMs are of particular concern because the majority (about 58%) of them are psychoactive drugs, such as benzodiazepines, opioids, antidepressants, and first-generation antihistamines [7], which are known to be associated with increased risks of crash involvement and culpability [1,11,12,33,34,35,36]. Results of the present study provide empirical evidence that use of PIMs by older drivers, in particular use of multiple PIMs, could impair their safety performance. 

Although many studies have examined the association of chronic medical conditions with crash risk, it remains a challenge to tease out the effect of specific medications from the effect of specific medical conditions. Given that the prevalence of multiple chronic medical conditions and polypharmacy use in the older driver population is over 90% [20,32], it is sensible to prioritize research using composite measures of comorbidities and medications, such as the Charlson Comorbidity Index score [37], PIMs [6], and Drug Burden Index [38], over studies of individual disease and medication in relation to driving safety in older adults.

In spite of notable strengths, such as the large sample size, comprehensive medication data and objectively and prospectively collected driving data, this study is limited by the observational study design and the proxy measure of driving safety. The prospective cohort design allows us to rigorously assess the association between PIM use and the risk of hard braking events, but makes it difficult, if not impossible, to infer causality. Moreover, the outcome measure in our study, hard braking event, is a proxy measure of driving safety. As a surrogate measure of driving safety, the incidence of hard braking events has been correlated to many contributing factors for crashes and is known to increase the reliability of risk estimation [21]. However, it should not be viewed as a substitute for crash risk before its validity for measuring crash risk is adequately established. Data on police-reported crashes are included in the LongROAD project. Because of the low incidence rate of motor vehicle crashes, it will take several more years of follow-up to accumulate enough crash records for adequately powered statistical analysis. Finally, the study sample is not nationally representative and thus the findings of this study may not be generalizable to the general older driver population in the United States. Compared to the general older driver population, participants of the LongROAD project are overrepresented by non-Hispanic white drivers and socioeconomically advantaged drivers as indicated by education attainment and annual household income [18].

## 5. Conclusions

Our study indicates that PIM use is associated with a significantly increased risk of hard braking events in a dose-response fashion in older drivers. In light of the well-documented adverse health consequences associated with PIM use in older adults, results of this study provide further impetus to implement effective intervention programs to reduce the prescription and use of PIMs in older adults. 

## Figures and Tables

**Table 1:** Incidence rates of hard braking events per 1000 miles by demographic characteristics, the Longitudinal Research on Aging Drivers (LongROAD) Study.

Variable	Number of Drivers *	Total Miles	Number of Hard Braking Events	Incidence Rate/1000 Miles	95% Confidence Interval	
Age, years						
65–69	1216	29,229,044	32,190	1.10	1.09	1.11
70–74	1019	23,158,476	27,148	1.17	1.16	1.19
75–79	697	13,483,350	17,076	1.27	1.25	1.29
Gender						
Male	1374	33,555,087	38,107	1.14	1.12	1.15
Female	1558	32,315,783	38,307	1.19	1.17	1.20
Race/Ethnicity						
White, non-Hispanic	2511	57,291,724	63,324	1.11	1.10	1.11
Black, non-Hispanic	207	4,480,516	5908	1.32	1.29	1.35
Other	210	4,027,178	7027	1.74	1.70	1.79
Marital Status						
Married	1846	43,593,440	46,824	1.07	1.06	1.08
Non-married	1059	21,745,043	28,835	1.33	1.31	1.34
Education						
High school or less	325	6,670,801	7034	1.05	1.03	1.08
Associate’s degree	712	16,292,645	20,246	1.24	1.23	1.26
Bachelor’s degree	684	15,192,134	17,964	1.18	1.17	1.20
Advanced degree	1203	27,553,763	31,012	1.13	1.11	1.14
Annual Household Income						
<$49,999	757	15,207,892	19,841	1.30	1.29	1.32
$50,000–$79,999	708	16,481,447	16,469	1.00	0.98	1.02
$80,000–$99,999	423	10,607,189	11,583	1.09	1.07	1.11
≥$100,000	938	21,406,285	26,120	1.22	1.21	1.24
Urbanicity						
Urban	2134	4,3714,100	60,976	1.40	1.38	1.41
Suburb/Rural	798	22,156,770	15,438	0.70	0.69	0.71
PIM use						
Yes	542	11,038,815	14,359	1.30	1.28	1.32
No	2390	54,832,055	62,055	1.13	1.12	1.14

* Totals within variables may vary due to missing data.

**Table 2:** Estimated incidence rate ratios and 95% confidence intervals of hard braking events from the multivariable negative binomial model according to potentially inappropriate medication (PIM) Use, race/ethnicity, marital status and urbanicity, the Longitudinal Research on Aging Drivers (LongROAD) Study.

Variable	Incidence Rate Ratio	95% Confidence Interval	
PIM use			
No	1.00		
Yes	1.10	1.01	1.20
Race/Ethnicity			
White, non-Hispanic	1.00		
Black, non-Hispanic	1.00	0.88	1.15
Other	1.38	1.21	1.57
Marital Status			
Married	1.00		
Non-married	1.21	1.12	1.29
Urbanicity			
Urban	1.00		
Rural/Suburban	0.51	0.48	0.56

## Data Availability

Restrictions apply to the availability of these data. Data are available from the author with permission from the AAA Foundation for Traffic Safety and upon execution of a data use agreement.
